# Christmas in War-Time

**Published:** 1916-12-16

**Authors:** 


### The Hospital

The Workers' Newspaper of Administrative Medicine and Institutional Life, Administration, National Insurance and Health.

No. 1593, Vol. XLI. SATURDAY, DECEMBER 16, 1916. PRICE ONE PENNY

### CHRISTMAS IN WAR-TIME.

Christmas Day, 1916, will be the first Christmas which the present generation of Englishmen have yet had to face in the heart of a great war. True, this war began in August 1914, but its vastness and England's unpreparedness, with other causes, have combined to keep back a full sense of the pressure and horrors of a vast international war until the present moment. To-day there is hardly a family in Great Britain where several of its members have not been or are not actively engaged in the war. Family life has been interfered with and largely broken up or destroyed. When in the history of our nation and Empire has there heretofore been a time which drew the women from the homestead and scattered them as busy workers engaged in all kinds of occupations, which five years ago would, for the most part, have been regarded as unsuitable and impossible for members of the weaker sex to follow? Even the children are so embraced by the war that every play-room and nursery has, as the centre of attraction, many kinds of weapons, war material, and fighting requisites. Further, the atmosphere, like the inhabitants of Great Britain, has entirely changed. Everything is different; and the great problem presented is, What will actually happen when war is ended?

In circumstances like these, what is the possible and proper attitude of mind this Christmastide, 1916, towards friends, family connections, children, one's neighbours, and dependants? Taking them altogether, the spirit of the times and the general feeling of the population is undoubtedly not to give a multitude of presents. The times are out of joint. Economy is enforced and rendered necessary by war conditions. Show and display offend the taste of all classes of people who properly apprehend the awfulness of the war, and its dire consequences to our youth and manhood, their families, comrades, and friends. Heavy taxation and ever-rising prices, even for the simplest commodities, render essential, care in expenditure and self-denial in purchases, with an enforced reduction of personal outlay to the lowest minimum. Nevertheless, the overwhelming feeling is to cultivate cheerfulness, encourage hope, and continue constant in prayer. So the will to do good deeds, to exhibit genuine kindness and ever-thoughtful consideration for all less fortunate than ourselves, whose lives touch ours in the daily round, was never so general, constant, or so all-embracing as Christmas finds it this year. The general cheerfulness promises that Christmas Day will be one of the heartiest and most enjoyable that the children of England have ever spent. The child is father to the man, and the man and the woman this year, who are blessed with children, have often had to learn from bereavement and the removal of their nearest and dearest, how to suffer. The heart yearns for an outlet, and the children come with tenfold blessings this year, because with them there is the supreme gift of merry minds and joyous hearts, that bring an atmosphere of supreme blessedness into the family gatherings everywhere. So, whatever else may be given up, it is perfectly certain that the children must be thought of and cared for with special devotion on the part of the parents, who should be moved to give time and thought to the arrangements for Christmas, that they may provide, adequately, innocent pleasure and uplifting spirits. The resolve to adopt this course on the part of parents cannot fail to bring them, not only relief from the pains and trials of the past, but an inspiring, hopeful peep into the future.

For the rest, two things, it seems to us, remain which will be worth doing with care and energy, on the first Christmas of the Great War. Let us each remember our old friends, wherever they be, and make a point of writing each a little note of sympathy and good will. It is remembrance, in times like these, which gives the touch of nature that makes the whole world kin. So, whatever claims there may be upon time and energies, let no one fail to renew old friendships, or to sweeten and cement them by a few spoken or written words of heartfelt greeting on Christmas Day.

So far, we have treated this war Christmas as an occasion which must deeply affect the personal life, circumstances, and occasions of every man and woman in the land. But outside these endearing ties there remains the responsibility of our duty towards dependants and those less fortunate than ourselves, with the further duty and privilege of remembering our warriors who are lying wounded in some hospital, the large number of munition and other workers who are in the same predicament from zealous services given in defence of their country, and the civilian population who are mainly dependent on our voluntary hospitals in the hour of sickness and accident. For those dependent upon us, and these less fortunate neighbours of ours, it will be our high privilege, not only to give something, but to expend thought upon the character and nature of each gift. Each present should be adequate and adapted to meet in the tenderest, kindest, and most thorough way, the needs of those it is our privilege to help. Whether such gifts should be anonymous, or not, must be left to the feeling of every donor in the circumstances of each case.

We have already pointed out that, although the expenditure of most hospitals has increased, a right spirit of patriotism should have restrained personal expenditure in all possible directions. And in the result there should be left a sum available which the possessors will be free this year to devote to relieve the sick and provide, in God's mercy, for the recovery of health and strength by those inmates of our hospitals who have suffered directly or indirectly from the war. That means practically the inmates of every hospital throughout the country. 1916 has been a very anxious and arduous year for hospital managers everywhere. On pages 229 to 233, will be found the names of a number of institutions to which we would direct the special attention of those able to give substantial sums this Christmas for the relief of the sick. Gifts of all kinds to the hospital nearest to our home will be welcome. Indeed, we heartily wish that the spirit of apprehension and the blessing attaching to personal service may make Christmas Day 1916 memorable in history, on account of the number and variety of separate donations and gifts, which each hospital will receive. There is no place on earth to compare with the inside of a hospital, when everybody connected with the staff and many of its friends give themselves up, throughout Christmas Day, to the personal service of endeavouring to bring peace and goodwill, forgetfulness of suffering, injury, loss, and an awakening to the Spirit of Christ; into every heart. Wherever this is accomplished, there will be an expression of thankfulness to Almighty God for His manifold mercies, to every family in the land.

